# Epidemiological characteristics and importation patterns of imported dengue fever in southwest border regions of China

**DOI:** 10.1371/journal.pntd.0014446

**Published:** 2026-06-22

**Authors:** Jiaxin Hao, Yong Shen, Huakun Xu, Fuying Guo, Dongsheng Ren, Tian Ma, Shanshan Song, Ruifang Song, Noksrikeaw Treenate, Qing Zhen, Zhe Wang, Tiejun Shui, Xiangyu Yan

**Affiliations:** 1 School of Disaster and Emergency Medicine, Tianjin University, Tianjin, China; 2 Department of Epidemiology and Biostatistics, School of Public Health, Jilin University, Changchun, Jilin, China; 3 Xishuangbanna Dai Autonomous Prefecture People’s Hospital, Jinghong, Yunnan, China; 4 The People’s Hospital of Lincang, Lincang, Yunnan, China; 5 National Key Laboratory of Intelligent Tracking and Forecasting for Infectious Diseases, National Institute for Communicable Disease Control and Prevention, Chinese Center for Disease Control and Prevention, Beijing, China; 6 Key Laboratory of Medical Rescue Key Technology and Equipment, Ministry of Emergency Management, Tianjin, China; 7 General Administration Office, Chinese Center for Disease Control and Prevention, Beijing, China; 8 Yunnan Center for Disease Control and Prevention, Kunming, Yunnan, China; Western Carolina University, UNITED STATES OF AMERICA

## Abstract

Dengue fever is a global public health issue of international concern. The southwestern border regions of China (Yunnan Province) are an important gateway for imported dengue fever cases in China. This study aims to investigate the importation patterns of imported dengue cases in Yunnan Province to support global dengue prevention efforts. This study examined imported dengue cases in Southwestern border regions of China, comparing actual and predicted cases via a counterfactual model under COVID-19 measures. Social network analysis was used to identify the importation pattern. From 2012 to 2023, 6,205 imported dengue cases were reported, making up 20.2%. The main sources were Southeast Asian countries: Myanmar (86.6%), Laos (5.8%), and Cambodia (4.9%). During the COVID-19 pandemic, imported cases dropped dramatically when borders closed, with predicted case counts being approximately 16.2-fold, 315.3-fold, and 12.3-fold higher than observed counts from 2020 to 2022, respectively, reflecting the sharp reduction in cross-border mobility during this period. In 2023, observed cases exceeded predictions by 45.0%. The cumulative social network identified three transmission communities centered around Kunming and Xishuangbanna in China, and Myanmar. The community centered in Myanmar had a bridging closeness centrality of 3.67, mainly connecting to Chinese border cities like Dehong (3.7) and Lincang (3.7). The Xishuangbanna community had a centrality of 3.4, primarily linking to Laos (3.4). The Kunming community had the highest centrality at 4.4, connecting mainly to non-border countries including Thailand (4.0), Cambodia (4.3) and Malaysia (2.8). Imported dengue cases in China’s southwest border regions exhibit a dual-center pattern: land-border cases cluster in cities adjacent to Myanmar and Laos, while air-border cases concentrate in Kunming. Future efforts should enhance port monitoring and cross-border cooperation.

## Author summary

Dengue fever is a mosquito-borne disease spreading globally. China’s Yunnan Province, which shares long borders with Myanmar, Laos, and Vietnam, serves as a major gateway for imported dengue cases. We analyzed 12 years of surveillance data (2012–2023) to understand where these imported cases originate and how they enter the region. We found that over 6,000 cases (20% of the total) were imported, with the vast majority coming from Myanmar (87%), followed by Laos and Cambodia. During the COVID-19 pandemic, imported cases dropped dramatically when borders closed, confirming that human movement drives dengue spread. Our analysis revealed two distinct importation patterns: land border crossings to cities adjacent to Myanmar and Laos, and air travel to the provincial capital Kunming. These findings highlight the need for tailored prevention strategies—strengthening surveillance at both land borders and airports. Enhanced cross-border cooperation with neighboring countries is essential to reduce imported dengue risk and protect communities in this vulnerable border region.

## Introduction

Dengue fever (DF) is an acute infectious disease caused by the dengue virus (DENV) and is transmitted through the bites of *Aedes albopictus* and *Aedes aegypti mosquitoes.* Dengue virus belongs to the Flaviviridae family and is divided into four serotypes (DENV-1 to DENV-4), all of which can cause human infections [1]. It is the fastest-spreading, most widely distributed, and most prevalent mosquito-borne viral disease in the world [2,3].

Over the past few decades, the global incidence of dengue fever has increased sharply. More than 128 countries have reported dengue infections [4]. Epidemiological studies have shown that approximately 3.97 billion people are at risk of DENV infection, mainly in tropical and subtropical regions [5]. Driven by globalization, climate change, urbanization, and population movement, dengue fever can easily spread from tropical regions to other geographical areas, causing outbreaks in nonendemic areas. Asia has been a high-incidence area for dengue fever for more than 30 years, accounting for 75% of the global disease burden. The main endemic areas are Southeast Asian countries, such as Myanmar, Thailand, and Malaysia [6]. China’s southwestern border, represented by Yunnan Province, borders dengue-endemic regions in Southeast Asia and South Asia, with long borders and numerous ports of entry (S1 Fig). Unlike Southeast Asian countries where dengue is year-round endemic, mainland China experiences seasonal dengue outbreaks primarily initiated by imported cases, with local transmission occurring in specific seasons and regions where competent vectors are present [7]. Yunnan Province, as an important province on China’s southwestern frontier, is one of the core provinces for China’s participation in the Greater Mekong Subregion Economic Cooperation (GMS). In recent years, cooperation between Yunnan Province and the Mekong countries in the fields of trade, investment, and infrastructure construction has continuously increased, with frequent interactions. These unique geographical and social factors have made Yunnan Province the province with the highest number of imported dengue cases in China, accounting for 40.9% of the country’s imported dengue cases [8–9]. In 2015, 2017, and from 2019 to 2023, Yunnan Province had the highest incidence rate of dengue fever in China, with an average annual incidence rate of 5.92 per 100,000 people [10].

In 2013, Xishuangbanna Prefecture and Dehong Prefecture in Yunnan Province, located on the China–Myanmar and China–Laos borders, experienced significant local dengue fever outbreaks triggered by imported cases for the first time [11]. In 2019, the epidemic areas of dengue fever in Yunnan Province continued to expand, and some regions experienced multiple outbreaks of dengue fever caused by imported cases [12]. At the beginning of 2020, in response to the COVID-19 pandemic, China began to implement strict lockdowns and entry–exit controls at border ports [13]. After the end of the COVID-19 pandemic, China lifted the lockdowns at border ports. As a result, China is once again facing the challenge of imported dengue fever outbreaks. However, previous research on the sources and patterns of imported dengue cases in China’s southwestern border regions has been relatively insufficient. Therefore, the aims of this study are threefold. First, we describe the status of imported dengue cases in Yunnan Province, China, from 2012 to 2023. Second, the impact of COVID-19 control measures on dengue fever was assessed via a counterfactual model. Third, we systematically analyze the sources and patterns of imported dengue cases through social network analysis, providing decision-making support for dengue fever prevention and control.

## Methods

### Ethical approval statement

This study was approved by the Research Ethics Committee of the Yunnan Center for Disease Control and Prevention (2023‐19). The research ethics committee waived informed consent because the study did not involve identifiable personal information.

### Data sources

Individual-level data for all dengue fever cases that occurred in Yunnan Province, China, from January 2012 to December 2023 were obtained from the China National Notifiable Disease Surveillance System (CNNDS). The diagnosis of dengue fever was based on the diagnostic criteria guidelines issued by the Chinese Center for Disease Control and Prevention (CDC). The anonymization of all case data used in this study made it impossible to disclose personal identities.

Our analysis included all clinically and laboratory-confirmed dengue fever cases in Yunnan Province. Overseas imported dengue cases were defined based on travel history within 14 days prior to symptom onset, not nationality. Cases were classified as imported if the patient had visited a dengue-endemic country or region within 14 days before disease onset, in accordance with the Exit and Entry Administration Law of the People’s Republic of China. This classification applies to both Chinese citizens (including those engaged in cross-border trade or labor) and foreign nationals. The base maps of Asia and Yunnan Province used for the study were obtained from the Institute of Geographic Sciences and Natural Resources, Chinese Academy of Sciences (http://www.resdc.cn/).

### Statistical analysis

#### Descriptive statistical analysis.

This study calculated the proportion of imported cases from each country and created a trend chart for the top 5 countries with the most imported cases. We also mapped the distribution of cases in Yunnan Province via ArcGIS 10.8.2 and created imported pathways of dengue fever cases from other countries to various prefectures/cities in Yunnan Province from 2012 to 2023 using Tableau Desktop.

#### Construction of the counterfactual model.

To assess the impact of COVID-19 control measures on dengue fever, we constructed a counterfactual model. The peak number of imported dengue fever cases usually occurs in November and December each year. Given this periodicity, an optimal Autoregressive Integrated Moving Average (ARIMA) model (*p*, *d*, *q*) (*P*, *D*, *Q*)*s* for dengue fever was established on the basis of the monthly number of imported dengue fever cases from January 2012 to December 2019. This model was used to predict the number of dengue cases during the COVID-19 pandemic period (January 2020-December 2023). The predicted case numbers were then compared with the actual reported case numbers during the same period.

The parameters *p* and *P* are the autoregressive term and seasonal autoregressive term, respectively; *d* and *D* are the respective nonseasonal difference and seasonal difference terms; *q* and *Q* are the moving average term and seasonal moving average term, respectively; and *s* is the periodic term [14]. The model predicted the number of dengue fever imported cases in the presence of the COVID-19 pandemic, which was also called the counterfactual model27 [15]. Specifically, using the  auto.arima ()  function from the forecast package in *R*, the ARIMA model was built on the basis of the training set, and an optimal set of parameters was automatically chosen by comparing the combined spectrum of parameters according to the rule of the minimum Akaike information criterion or Bayesian information criterion [16]. Model diagnostics included Ljung-Box test for residual white noise and ACF/PACF plots. Forecast accuracy was measured by MAE, RMSE and sMAPE, as MAPE was incalculable for zero-inflated data. The absolute percent error (APE) was calculated on the basis of the actual and predicted monthly number of cases ([actual number of cases−predicted number of cases]/actual number of cases) [17]. Because this metric can produce values exceeding ±100% when observed cases approach zero, deviations during the COVID-19 period are reported as the ratio of predicted to observed cases for clarity. The data were analyzed via R version 4.4.1.

#### Social network analysis.

***Construction of social networks*:** A social network is a complex structure composed of individuals and their interrelationships, reflecting interactions, connections, and information flow among individuals [18]. The graphical representation of a social network consists of nodes and edges connecting these nodes. In this study, nodes represented the countries or regions that imported dengue fever cases into Yunnan Province from abroad, as well as various cities in Yunnan Province from 2012 to 2023. Edges represent the presence of imported links, and the weights of the edges indicate the number of cases. The greater the weight is, the more cases are imported. The dynamic temporal-sliced social network was based on a static network with added temporal information. It involves segmenting the dynamic network along the temporal dimension [19]. For the period from 2012 to 2023, the time series data were divided into 12 temporal slices, with each slice representing the network state of imported dengue cases within one year.

***Analysis of the geospatial characteristics of social networks*:** The connections between nodes in social networks are often not uniform. There exist some subnetworks with particularly dense local relationships within social networks, which we refer to as communities [20]. Within communities, the connections between nodes are relatively dense, whereas connections between different communities are relatively sparse. Different communities are able to form a cohesive network because certain key nodes act as bridges connecting two communities. These nodes are referred to as bridge nodes [21]. In this study, the Algorithm for Spin Glass Community Discovery was used to divide the constructed social network of imported dengue fever cases between domestic and international regions into different transmission communities. After community detection was complete, the bridging closeness centrality measure was calculated to assess the connectivity of a node to other communities. Bridging closeness centrality reflects the average distance from a particular node to nodes in other communities [21,22]. Let a network with *V* vertices and *E* edges be denoted as G(V, E). Let *C* be a community in the network *G*, where C∈G; *a* is a vertex within community *C*, where a∈C; and *b* is a vertex outside of community *C*, where b∉C. Let N(a) represent the set of vertices connected to *a*, and let $w_{k}$ represent the weight of an edge, where $\;w_{ab}\in E$. Let $P_{ab}\;$ be the shortest path between *a* and *b*, with $E(P_{ab})\;=\;\{e_{\text{1}},\ldots ,e_{k},\ldots ,e_{n}\}$. The specific algorithm for vertex *a* is as follows:


$$\begin{array}{c} bridge\;closeness=\frac{\left|V-C\right|}{\sum_{b\in (V-C)}\sum_{e_{k}\in E(P_{ab})}\frac{\text{1}}{w_{k}}} \end{array}$$

The greater the bridging closeness centrality is, the shorter the average distance from node a to nodes in other communities, which indicates that the node is more important in connecting different communities.

Dynamic temporal slices of the imported dengue fever social network in Yunnan Province were constructed via R 4.4.1 and Gephi software.

### Ethics statement

This study was approved by the Research Ethics Committee of the Yunnan Center for Disease Control and Prevention (2023‐19). The research ethics committee waived informed consent because the study did not involve identifiable personal information.

## Results

### Number of dengue fever cases

**Fig 1:**
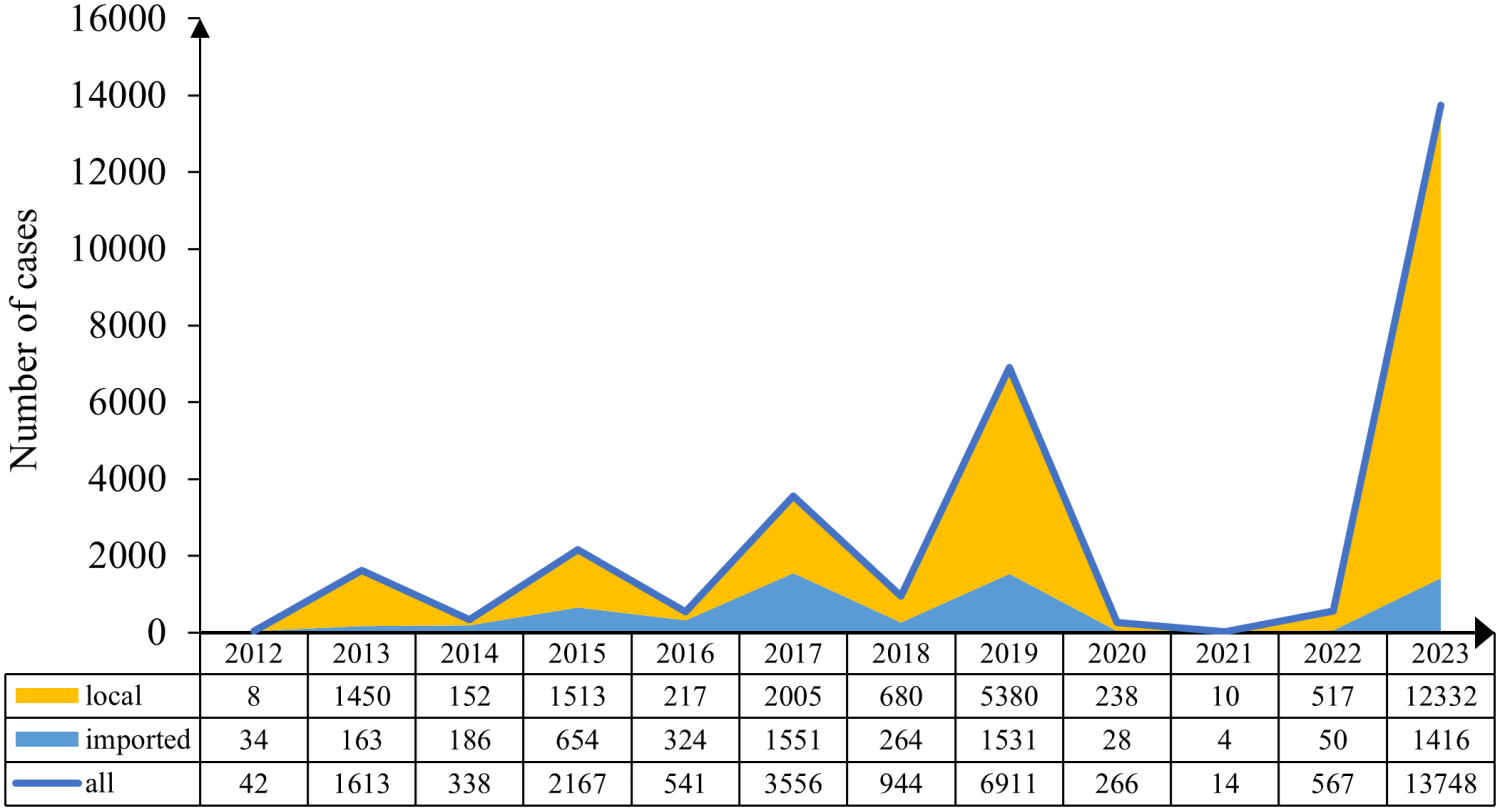
Trends chart of dengue fever cases from 2012 to 2023. Notable magnitude differences exist between imported case peaks and local transmission peaks, particularly evident during high-incidence years.

From 2012 to 2023, Yunnan Province reported a total of 30,707 cases of dengue fever, of which 6,205 (20.2%) were imported cases and 24,502 (79.8%) were locally acquired cases. Between 2012 and 2023, case numbers increased year-over-year in all odd-numbered years except 2021, decreased year-over-year in all even-numbered years except 2022, and reached particularly pronounced peaks in 2019 and 2023, increasing by 632% and 2324%, respectively (Fig 1). The trend of imported cases followed a similar pattern. The number of local cases fluctuated significantly, with pronounced peaks in 2013, 2015, 2017, 2019, and 2023.

### Geographical distribution of dengue fever cases

**Fig 2:**
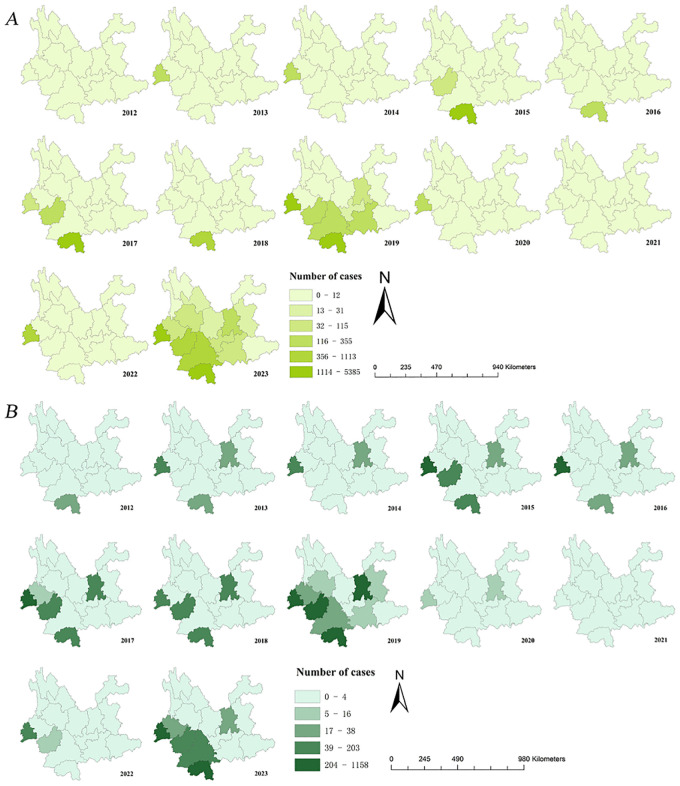
Geographical distribution of dengue fever cases (A) Geographical distribution of local dengue fever cases in Yunnan Province from 2012 to 2023. **(B)** Geographical distribution of imported dengue fever cases in Yunnan Province from 2012 to 2023 (https://www.resdc.cn/DOI/DOI.aspx?DOIID=121).

From 2012 to 2023, the geographical distribution of local dengue fever cases in Yunnan Province significantly changed. The number of affected prefectures/cities increased from 3 to 16. Overall, the top five prefectures/cities with the highest cumulative number of local cases were Xishuangbanna, Dehong, Lincang, Puer, and Kunming, with 14,220, 6,232, 1,655, 1,277, and 497 cases, respectively. These prefectures/cities accounted for 58.0%, 25.4%, 6.8%, 5.2%, and 2.0% of the total number of local cases in Yunnan Province, respectively. The corresponding incidence rates were 12.94, 6.37, 0.98, 0.77, and 0.08 per 10,000 population, respectively. Prefectures/cities with annual local case numbers exceeding 1,000 cases gradually became concentrated in the southwestern and southern regions of Yunnan Province, particularly in border areas adjacent to Myanmar, Laos, and Vietnam. Dengue fever cases appeared to show potential spatial clustering, with apparent concentrations in specific urban areas (based on visual inspection of Fig 2). From 2013 to 2017, cases were mainly concentrated in Dehong, Lincang and Xishuangbanna, Yunnan Province, China. From 2019 to 2023, the distribution of cases became more widespread across Yunnan Province, with a large number of dengue fever cases distributed in several cities, including Kunming, Puer and Yuxi (Fig 2).

From 2012 to 2023, imported dengue fever cases in Yunnan Province exhibited significant spatial heterogeneity. The number of prefectures/cities with imported cases increased from 3 to 15. The imported cases are concentrated mainly in Kunming in Yunnan Province, and the southwestern border regions of Yunnan Province, with the scope of the centers in the southwestern border regions expanding. Overall, the top five prefectures/cities in Yunnan Province, with the highest cumulative number of imported cases were Dehong, Xishuangbanna, Lincang, Kunming, and Puer, with 2,851, 1,657, 921, 560, and 86 cases, respectively. These prefectures/cities accounted for 45.9%, 26.7%, 14.8%, 9.0%, and 1.4%, respectively, of the total number of imported cases in Yunnan Province (Fig 2).

### The main source of imported dengue fever cases

**Fig 3:**
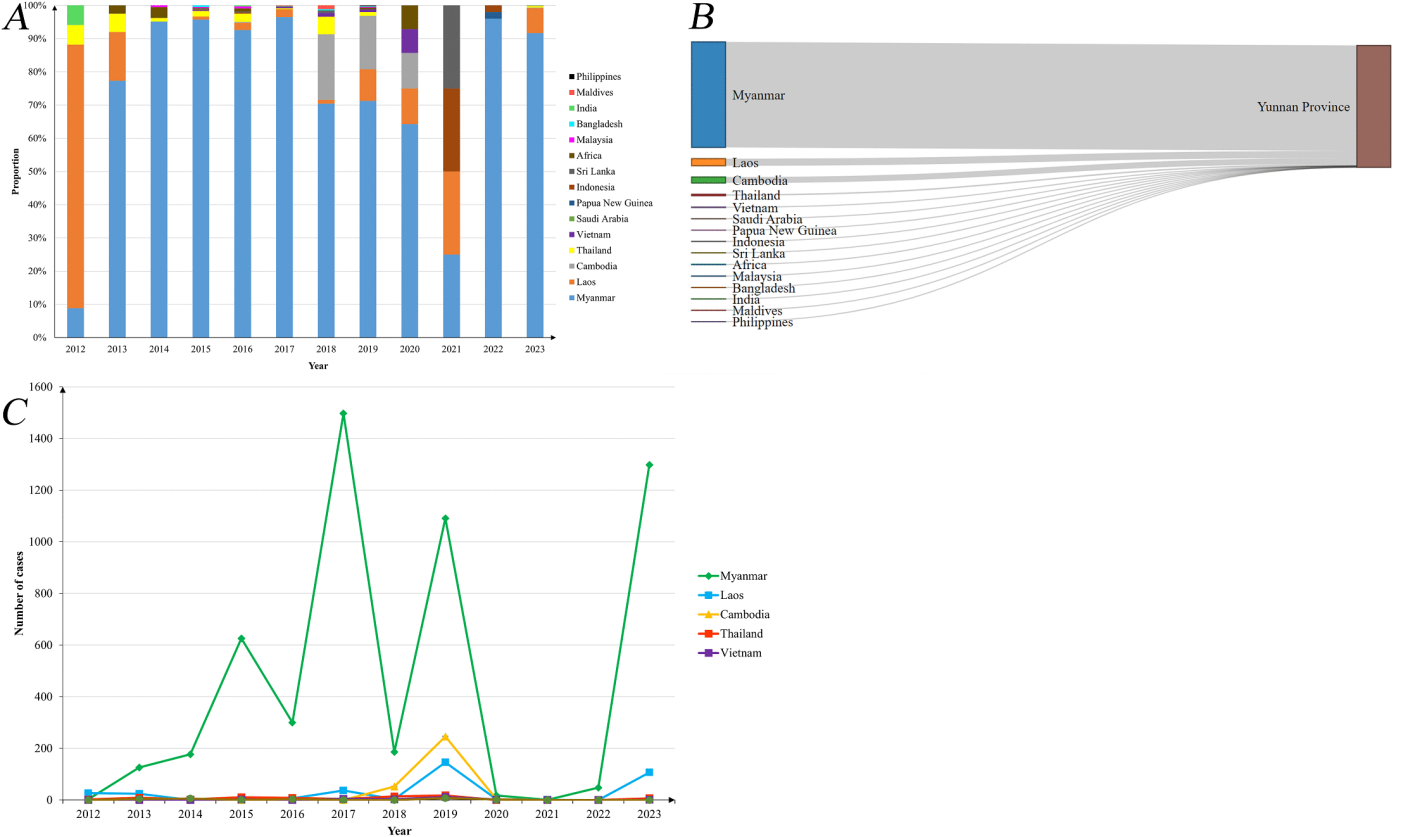
Countries of origin for imported dengue fever cases in Yunnan Province, China (A) Proportion of countries of origin for imported dengue fever cases in Yunnan Province, China, from 2012 to 2023. **(B)** Source countries of imported dengue fever cases in Yunnan Province from 2012 to 2023. **(C)** The top 5 countries with the most imported dengue fever cases to Yunnan Province, China, between 2012 and 2023.

From 2012 to 2023, Yunnan Province reported a total of 6,205 imported dengue fever cases. The year with the highest number of imported cases was 2017, with 1,551 cases (S1 Table). During this period, imported cases in Yunnan Province were from 20 different countries, including Asia (Myanmar, Laos, Thailand, etc.), Africa (Nigeria, the Democratic Republic of the Congo, etc.), and Oceania (Papua New Guinea). Myanmar was the primary source country for imported dengue fever cases in Yunnan Province, with a total of 5,371 imported cases, accounting for 86.6% of all imported cases. Laos followed with 361 cases (5.8%), and Cambodia followed with 304 cases (4.9%) (Fig 3).

Importation patterns reflected travel volume and geographic proximity. The high case numbers from Myanmar likely reflect its extensive border with Yunnan and established cross-border mobility, highlighting the importance of regional connectivity in disease importation dynamics. Imported cases from Myanmar experienced pronounced peaks in 2017, 2019, and 2023, with 1,497, 1,091, and 1,298 cases, respectively. Other countries, such as Laos, Cambodia, Thailand, and Vietnam, had relatively fewer imported cases, but there were still some increases in certain years. In 2012, Laos was the country with the highest number of cases imported into Yunnan Province. In 2017 and 2023, Laos had the most imported cases to Yunnan Province, except for Myanmar. In 2019, Cambodia had the most imported cases to Yunnan Province, except for Myanmar (Fig 3).

### The effects of COVID-19 control measures on dengue fever

The optimal counterfactual model for dengue fever in Yunnan Province was ARIMA (1,1,1) (2,0,0)_12_(AIC = 1015.31, BIC = 1028.08). Model diagnostics confirmed residual white noise properties: Ljung-Box tests were non-significant at lag-12 (χ^2^ = 6.52, df = 8, p = 0.806) and lag-24 (χ^2^ = 30.02, df = 20, p = 0.184), and inspection of ACF/PACF plots revealed no significant autocorrelation (all coefficients within 95% confidence intervals). Residual mean was 4.71 (SD = 44.45), approximating zero, with Shapiro-Wilk test indicating non-normality (p < 0.001) driven by epidemic outliers during high-transmission seasons (S3 Fig).

**Fig 4:**
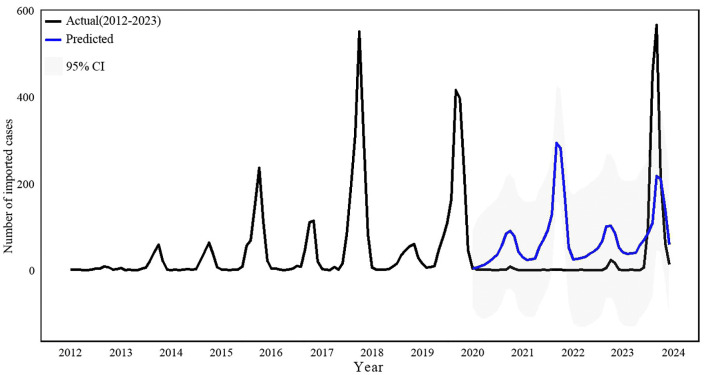
Comparison chart of predicted and actual imported dengue fever cases from 2020 to 2023.

During the COVID-19 pandemic, observed imported dengue cases were substantially lower than counterfactual model predictions. In 2020–2022, predicted case counts were approximately 16.2-fold, 315.3-fold, and 12.3-fold higher than the observed counts, respectively, reflecting the sharp reduction in cross-border mobility during this period. In 2023, observed cases exceeded predictions by 45.0% (Fig 4).

### Cross-border importation patterns and transmission communities

S3 Fig clearly illustrates the networks formed by different countries and cities, as well as the evolution of communities. From 2012 to 2023, the number of imported cases from countries other than Myanmar varied over time. From 2012 to 2014, the connections between nodes were relatively sparse and were concentrated mainly between a few locations, such as Kunming and Xishuangbanna in China and Myanmar. Myanmar had the highest bridging closeness centrality in 2012 and 2013, with values of 0.7 in 2012 and 5.3 in 2013. After 2014, connections between nodes increased gradually over time. For example, Xishuangbanna, Yunnan Province, China, had the highest bridging closeness centrality in 2015 and 2016, with values of 3.2 in 2015 and 2.2 in 2016, Cambodia had relatively high bridging closeness centrality in 2018 and 2019, with values of 2.80 in 2018 and 14.8 in 2019. The density of the network clearly increased gradually, increasing the complexity of the dengue fever importation network.

Additionally, some nodes maintained a high degree of connectivity throughout the entire period, and these nodes were likely key hubs in the dengue fever importation network, such as Kunming and Xishuangbanna in China. The bridging closeness centrality value showed that Kunming had a cumulative value of 4.4, making it the most central node in the network. It was also the most central node in multiple years. Xishuangbanna also had high network centrality, with a cumulative value of 3.4. The sustained high connectivity of these nodes was consistent with bridging closeness centrality.

**Fig 5:**
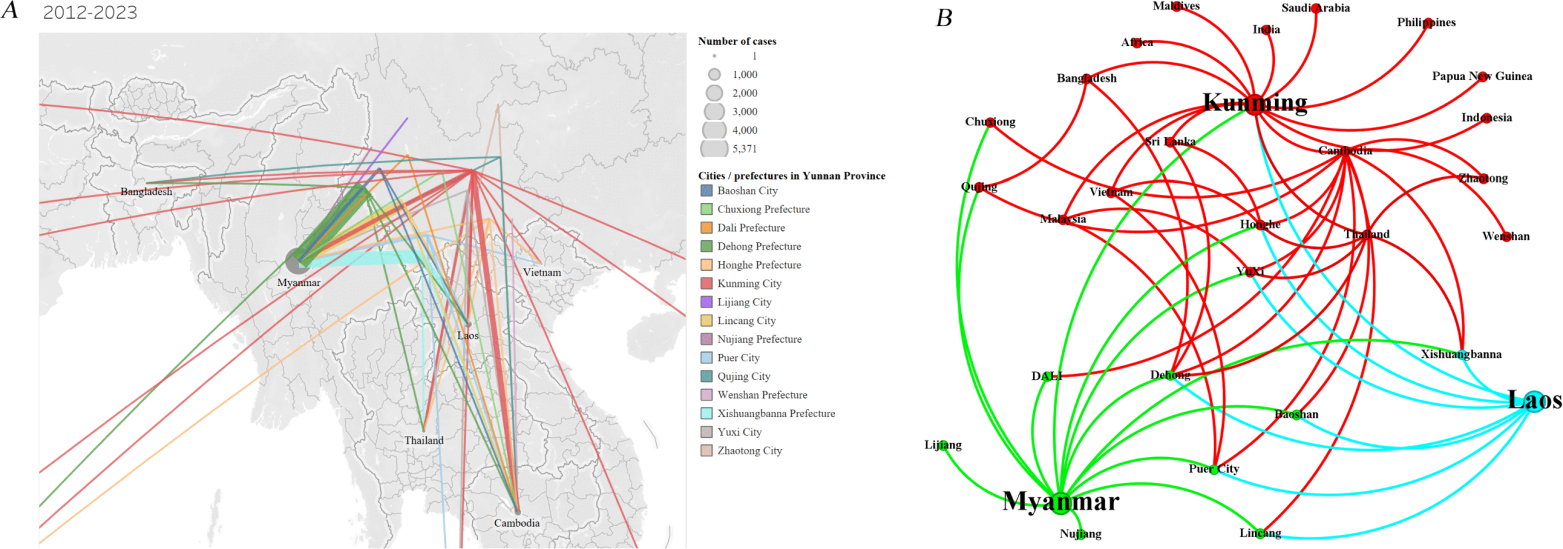
Cumulative social network diagram and pathway diagram of imported dengue fever cases from 2012 to 2023 (A) Dengue fever case pathway diagram. **(B)** Social network diagram of imported dengue fever cases (https://www.resdc.cn/data.aspx?DATAID=205).

There is a cumulative network displaying three distinct communities centered around Kunming and Xishuangbanna in China, and Myanmar (Fig 5). The community centered around Kunming is characterized by an air transport pattern, which mainly connects with countries that do not share a border with Yunnan Province, such as Thailand, Cambodia, and Malaysia. The community centered around Xishuangbanna Yunnan Province, China, is characterized by a land transport pattern, with the main connection with Laos. The community centered around Myanmar is also characterized by a land transport pattern, which mainly connects with Yunnan Province’s prefectures/cities that border Myanmar, such as Dehong and Lincang.

## Discussion

Dengue fever is considered an imported infectious disease in mainland China, as the country lacks natural endemic foci of DENV. While local outbreaks occur through secondary transmission initiated by imported cases (accounting for 79.8% of cases in this study), sustained indigenous transmission has not been established [23]. Therefore, imported cases are a key factor influencing local dengue fever outbreaks in China. Yunnan Province is located on the southwestern border of China and shares borders with Myanmar, Laos, and Vietnam. It has 20 national first- and second-class ports of entry and numerous border crossings, facilitating the frequent cross-border movement of local and large commercial and tourist flows [24].

In recent years, the Greater Mekong Subregion Economic Cooperation (GMS) has intensified cross-border trade, infrastructure development, and labor mobility between Yunnan and neighboring dengue-endemic countries such as Myanmar, Laos, and Thailand. This increased economic integration has facilitated frequent population movements, leading to the importation of dengue fever into China through infected travelers or virus-carrying *Aedes mosquitoes* [25]. From 2005 to 2020, China reported a total of 12,701 imported dengue fever cases. The majority of these cases were concentrated in Yunnan and Guangdong in China, accounting for 36.1% and 19.5% of the total imported cases, respectively. Among these, Ruili in Yunnan had the highest number of imported cases, with a total of 1,950 cases [26]. The results of this study also show that Yunnan Province in China is significantly affected by imported dengue fever cases from Myanmar and Laos. Dehong and Xishuangbanna, which border these two countries, are the two prefectures/cities with the highest number of imported cases in Yunnan Province. The continuous introduction of imported pathogens is a necessary condition for local outbreaks. However, the peak amplitude of local cases far exceeds that of imported cases, indicating that local transmission dynamics such as meteorological factors and population susceptibility may play a dominant role in epidemic amplification.

After 2020, the intensity of the dengue fever epidemic reported during the COVID-19 pandemic in China changed compared with the intensity reported during the previous decade. Compared with non-pandemic years, the dengue fever epidemic has decreased significantly [27]. This study compared imported dengue fever cases in Yunnan Province during the COVID-19 pandemic. We found that public health interventions for the COVID-19 pandemic have had a suppressive effect on imported dengue fever cases in Yunnan Province. To some extent, stricter public health measures can produce more significant effects. Travel restrictions and border closures could directly reduce the number of imported cases to the greatest extent, thereby significantly decreasing the local reported incidence rate and preventing outbreaks [28]. This also demonstrated that local epidemics are often triggered by imported cases. However, research has indicated that in regions where dengue fever cases are primarily imported, focusing on prevention at ports of entry and strengthening port management are effective means of preventing dengue fever. In contrast, in regions where dengue fever has been endemic for many years, such as Thailand and Pakistan, these measures are ineffective [29].

We note that vector distribution varies between source countries and Yunnan. *Aedes aegypti* and *Aedes albopictus* are more widely distributed and abundant in Myanmar and other endemic Southeast Asian countries, contributing to higher transmission intensity in these regions. This ecological gradient may contribute to higher importation volumes from areas with intense transmission. However, our analysis of annual-level trends inherently smooths seasonal variation, and the marked reduction in cases observed during 2020–2022 is consistent with nationwide trends reported by Zhai et al [30], who documented decreased dengue activity across Chinese mainland during this period. However, this reduction likely reflects the combined effects of border closures, internal mobility restrictions, reduced healthcare-seeking behavior, and diagnostic delays, rather than solely decreased importation. Our counterfactual model captures the aggregate impact of these measures but cannot disentangle the relative contribution of each factor. Future studies integrating vector surveillance data with human mobility metrics would provide a more comprehensive understanding of these complementary factors. Our counterfactual model captured the aggregate effect. However, such extreme measures carried substantial economic and social costs. They disrupted trade, impacted livelihoods, and strained healthcare resources, rendering them unsustainable for long-term control. As cross-border activities resumed under the Greater Mekong Subregion Economic Cooperation framework, sustainable strategies must shift from movement restriction to balanced approaches: enhanced surveillance, targeted vector control at ports of entry, and cross-border coordination through joint prevention mechanisms and information-sharing platforms, thereby mitigating disease risk without compromising economic cooperation.

Additionally, this study was limited by the lack of genomic data to confirm transmission chains, potential underreporting of mild cases, and inability to distinguish daily cross-border commuters from short-term travelers in border regions.

From 2012 to 2023, imported cases in Yunnan Province were from approximately 20 different countries. Among them, Myanmar had the highest number of imported cases, whereas Saudi Arabia and Papua New Guinea had the lowest. These findings underscore the significant role of cross-border importation in dengue epidemiology in Yunnan. Additionally, through our dynamic network analysis, over the 12 years, the countries that have contributed to the spread of dengue fever in Yunnan Province have formed three distinct importation clusters: one centered around Kunming in China, another around Xishuangbanna in China, and the third around Myanmar. This may be related to the modes of transportation used for transmission.

In Yunnan Province, which borders neighboring countries, a large proportion of imported cases are transmitted via land routes. Frequent trade and family visits among border residents made it difficult to control dengue fever. For example, Xishuangbanna in China, which borders Laos and Myanmar, has a well-developed tourism industry, and the main source of imported cases is Laos. Moreover, Xishuangbanna had a high cumulative betweenness centrality value of 3.4, indicating a high level of network centrality. Dehong and Lincang in Yunnan Province in China, which borders Myanmar, belong to the same community. Myanmar’s cumulative betweenness centrality value was as high as 3.7, which had a significant influence on the bordering cities in Yunnan Province. In contrast, prefecture/cities without international land borders, such as Kunming in Yunnan Province in China, were likely to have more imported cases via air travel [31]. Moreover, Kunming’s cumulative betweenness centrality value was 4.4, making it the most central node in the network. Kunming’s well-developed air transportation network and frequent international travel activities were associated with the highest volume of imported dengue cases, reflecting its role as a major entry point for international arrivals to Yunnan. Through a network community analysis of imported cases in Yunnan Province, it was revealed that the imported dengue fever cases in the region established a dual-center pattern of importation, primarily via land and air transport. For future prevention efforts, the following measures must be taken: First, inspection at borders and airports should be strengthened, air-imported cases to Kunming should be focused on, and intensive monitoring of flights from dengue-endemic areas should be conducted. Second, cooperation with neighboring countries should be strengthened, and epidemic information should be shared. The network communities focus on the main source countries for imported cases into Yunnan prefecture/cities, allocate disease control resources reasonably, and coordinate border control measures.

However, this study has several limitations. First, passive surveillance data are subject to reporting biases, including variations in healthcare-seeking behavior and diagnostic capacity changes over time. Second, network analysis is limited by administrative-level data aggregation; without genomic sequencing or contact-tracing data, we cannot establish transmission directionality or confirm that edges represent true epidemiological links rather than spatial proximity. Third, the 14-day travel history definition may misclassify daily cross-border commuters in border prefectures. Finally, underreporting of mild cases may underestimate true disease burden. Additionally, the COVID-19 period represented an unprecedented natural experiment with multiple simultaneous interventions [30]. While our counterfactual model quantifies the aggregate deviation from predicted trends, we cannot isolate the specific contribution of decreased importation from internal mobility restrictions, changes in healthcare-seeking behavior, diagnostic delays, or reporting disruptions.

## Conclusion

This study analyzed the epidemiological characteristics and importation patterns of imported dengue fever cases in Yunnan Province, China, from 2012 to 2023. Most imported cases originated from Myanmar, Laos, and Cambodia. Xishuangbanna, Dehong, and Kunming reported the highest numbers of imported cases.

Social network analysis identified three transmission communities: a Myanmar-centered community connecting to Dehong and Lincang via land borders, a Xishuangbanna-centered community linking to Laos via land routes, and a Kunming-centered community connecting to Thailand, Cambodia, and Malaysia through air travel. This dual-center pattern indicates that land-border importation clustered in cities adjacent to Myanmar and Laos, while air-border importation concentrated in Kunming. Kunming exhibited the highest bridging closeness centrality, followed by Myanmar and Xishuangbanna.

These findings suggest that prevention strategies should be tailored to distinct importation patterns. Land-border areas require strengthened port surveillance and cross-border cooperation with Myanmar and Laos, while Kunming requires intensified airport screening of flights from endemic areas. Establishing information-sharing mechanisms and coordinated prevention measures across transmission communities may reduce importation risk and protect communities in this border region.

## Supporting information

S1 Fig Geographical location of Yunnan Province. (A) Surrounding countries of Yunnan Province. (B) Prefectures/Cities in Yunnan Province (https://www.resdc.cn/data.aspx?DATAID=205).
(TIF)

S2 Fig Residual diagnostics of the fitted forecasting model. (TIF)

S3 Fig The imported dengue fever case pathway diagram and social network diagram from 2012 to 2023, where lines of the same color represent the same community. (A) The imported dengue fever case pathway diagram and social network diagram from 2012 to 2017 (B) The imported dengue fever case pathway diagram and social network diagram from 2017 to 2023 (https://www.resdc.cn/data.aspx?DATAID=205).
(TIF)

S1 Table Number of imported dengue fever cases from 2012 to 2023. (DOCX)

S1 Data Aggregated data underlying the study findings presented in the figures and tables. (DOCX)
