# Supplementary figures and images for: Epidemiological characteristics and importation patterns of imported dengue fever in southwest border regions of China
Source: PLoS Negl Trop Dis. 2026 Jun 22;20(6):e0014446. doi: 10.1371/journal.pntd.0014446 (PMC13336463; doi:10.1371/journal.pntd.0014446)

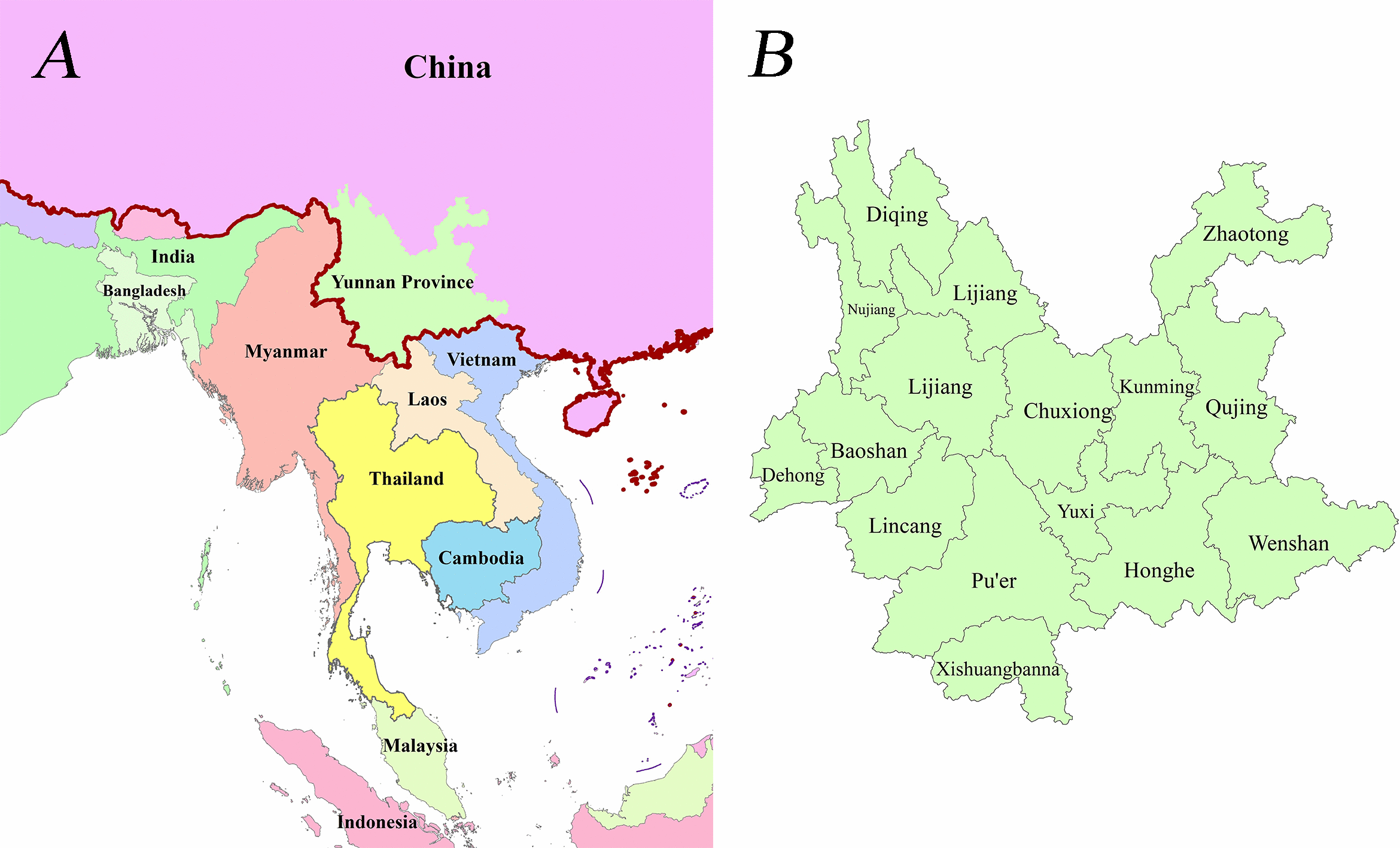

Supplement: S1 Fig — (A) Surrounding countries of Yunnan Province. (B) Prefectures/Cities in Yunnan Province (https://www.resdc.cn/data.aspx?DATAID=205). (TIF) [file pntd.0014446.s001.tif]

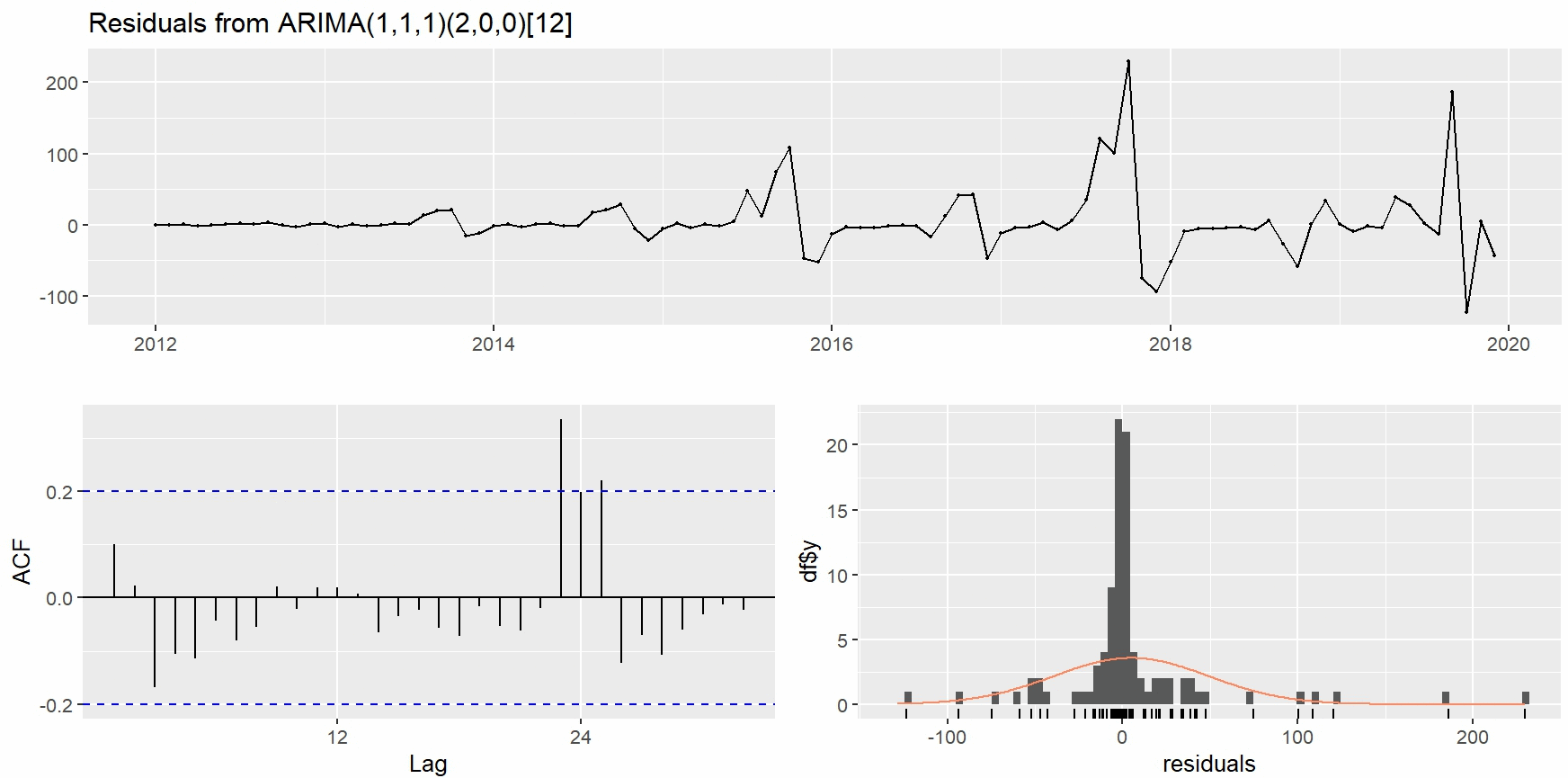

Supplement: S2 Fig — (TIF) [file pntd.0014446.s002.tif]

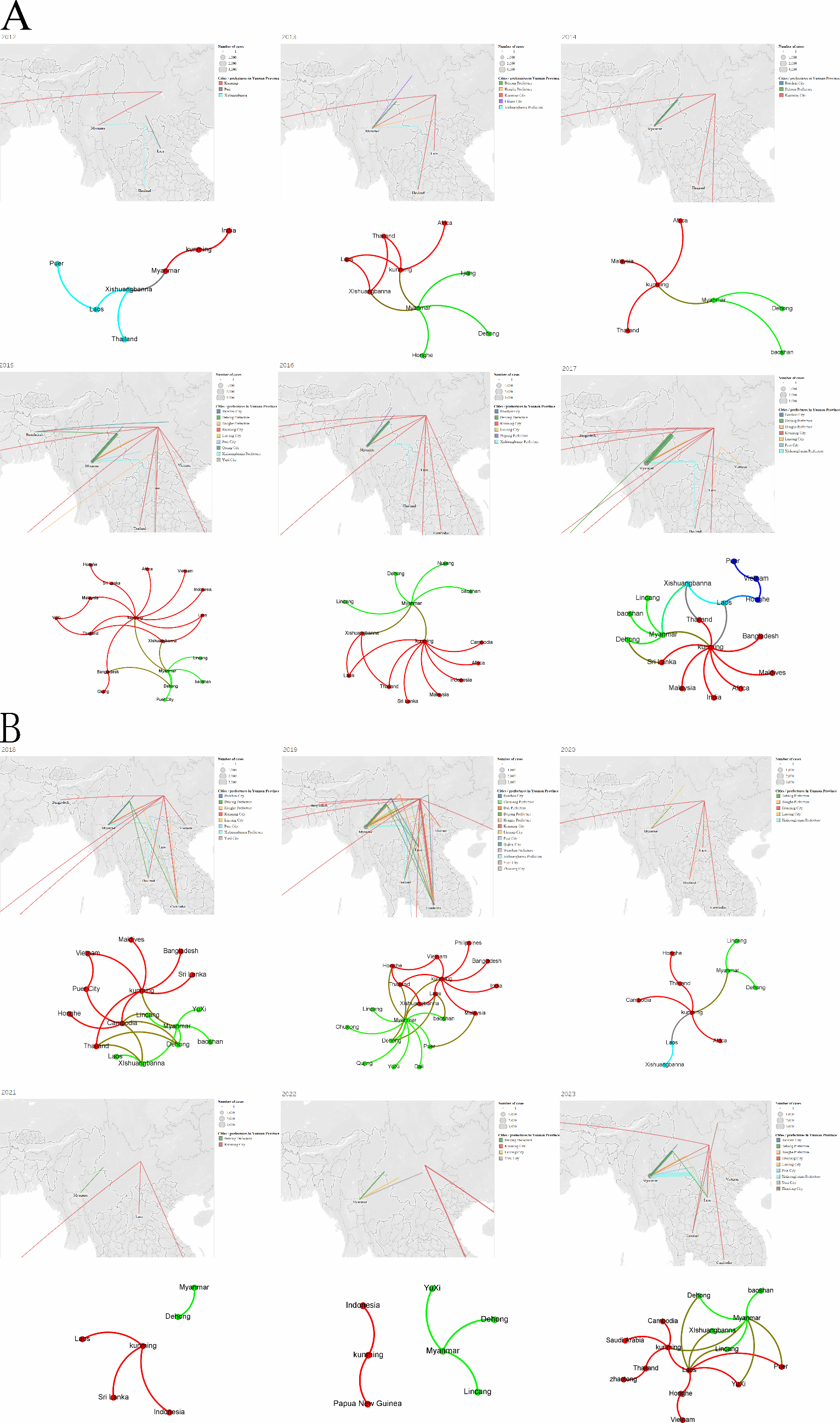

Supplement: S3 Fig — (A) The imported dengue fever case pathway diagram and social network diagram from 2012 to 2017 (B) The imported dengue fever case pathway diagram and social network diagram from 2017 to 2023 (https://www.resdc.cn/data.aspx?DATAID=205). (TIF) [file pntd.0014446.s003.tif]
