# Supplementary material for: Epidemiological characteristics and importation patterns of imported dengue fever in southwest border regions of China
Source: PLoS Negl Trop Dis. 2026 Jun 22;20(6):e0014446. doi: 10.1371/journal.pntd.0014446 (PMC13336463; doi:10.1371/journal.pntd.0014446)
Supplement: S1 Table — (DOCX) [file pntd.0014446.s004.docx]

Supplementary Table S1 Number of imported cases from 2012 to 2023

| Country | 2012  (N=24)  n (%) | 2013  (N=163)  n (%) | 2014  (N=186)  n (%) | 2015  (N=654)  n (%) | 2016  (N=324)  n (%) | 2017  (N=1551)  n (%) | 2018  (N=264)  n (%) | 2019  (N=153)  n (%) | 2020  (N=28)  n (%) | 2021  (N=4)  n (%) | 2022  (N=50)  n (%) | 2023  (N=1416)  n (%) | Total  (N=6205) |
| --- | --- | --- | --- | --- | --- | --- | --- | --- | --- | --- | --- | --- | --- |
| Myanmar | 3(8.8) | 126(77.3) | 177(95.2) | 626(97.5) | 300(92.6) | 1497(96.5) | 186(70.5) | 1091(71.3) | 18(64.3) | 1(25.0) | 48(96.0) | 1298(91.7) | 5371 |
| Laos | 27(79.4) | 24(14.7) | 0 | 6(0.9) | 7(2.2) | 37(2.4) | 3(1.1) | 146(9.5) | 3(10.7) | 1(25.0) | 0 | 107(7.6) | 361 |
| Cambodia | 0 | 0 | 0 | 0 | 1(0.3) | 0 | 52(19.7) | 246(16.1) | 3(10.7) | 0 | 0 | 2(0.1) | 304 |
| Thailand | 2(5.9) | 9(5.5) | 2(1.1) | 11(1.7) | 8(2.5) | 4(0.3) | 14(5.3) | 18(1.2) | 0 | 0 | 0 | 7(0.5) | 75 |
| Vietnam | 0 | 0 | 0 | 2(0.3) | 0 | 5(0.3) | 3(1.1) | 12(0.8) | 2(7.1) | 0 | 0 | 1(0.1) | 25 |
| Saudi Arabia | 0 | 0 | 0 | 0 | 0 | 0 | 0 | 0 | 0 | 0 | 0 | 1(0.1) | 1 |
| Papua New Guinea | 0 | 0 | 0 | 0 | 0 | 0 | 0 | 0 | 0 | 0 | 1(2.0) | 0 | 1 |
| Indonesia | 0 | 0 | 0 | 1(0.2) | 1(0.3) | 0 | 0 | 0 | 0 | 1(25.0) | 1(2.0) | 0 | 4 |
| Sri Lanka | 0 | 0 | 0 | 2(0.3) | 1(0.3) | 3(0.2) | 2(0.8) | 0 | 0 | 1(25.0) | 0 | 0 | 9 |
| Africa | 0 | 4(2.5) | 6(3.2) | 1(0.2) | 3(0.9) | 1(0.1) | 0 | 8(0.5) | 2(7.1) | 0 | 0 | 0 | 25 |
| Malaysia | 0 | 0 | 1(0.5) | 2(0.3) | 2(0.6) | 1(0.1) | 0 | 4(0.3) | 0 | 0 | 0 | 0 | 10 |
| Bangladesh | 0 | 0 | 0 | 3(0.5) | 0 | 1(0.1) | 1 | 2(0.1) | 0 | 0 | 0 | 0 | 7 |
| India | 2(5.9) | 0 | 0 | 0 | 1(0.3) | 1(0.1) | 0 | 1(0.1) | 0 | 0 | 0 | 0 | 5 |
| Maldives | 0 | 0 | 0 | 0 | 0 | 1(0.1) | 3(1.1) | 1(0.1) | 0 | 0 | 0 | 0 | 5 |
| Philippines | 0 | 0 | 0 | 0 | 0 | 0 | 0 | 2(0.1) | 0 | 0 | 0 | 0 | 2 |
