# Supplementary material for: Epidemiological characteristics and importation patterns of imported dengue fever in southwest border regions of China
Source: PLoS Negl Trop Dis. 2026 Jun 22;20(6):e0014446. doi: 10.1371/journal.pntd.0014446 (PMC13336463; doi:10.1371/journal.pntd.0014446)
Supplement: S1 Data — (DOCX) [file pntd.0014446.s005.docx]

**Supporting data of each figures in the manuscript**

Figure 2. Geographical distribution of dengue fever cases(A) Geographical distribution of local dengue fever cases in Yunnan Province from 2012 to 2023.

| City/Prefecture | 2012 | 2013 | 2014 | 2015 | 2016 | 2017 | 2018 | 2019 | 2020 | 2021 | 2022 | 2023 |
| --- | --- | --- | --- | --- | --- | --- | --- | --- | --- | --- | --- | --- |
| Kunming | 2 | 7 | 7 | 10 | 0 | 10 | 1 | 110 | 1 | 0 | 0 | 355 |
| Qujing | 0 | 2 | 2 | 0 | 0 | 0 | 0 | 6 | 0 | 0 | 0 | 23 |
| Yuxi | 0 | 0 | 0 | 0 | 0 | 0 | 0 | 17 | 0 | 0 | 0 | 80 |
| Baoshan | 0 | 2 | 2 | 2 | 0 | 6 | 0 | 2 | 0 | 0 | 0 | 115 |
| Zhaotong | 0 | 1 | 1 | 0 | 0 | 0 | 0 | 2 | 0 | 0 | 0 | 12 |
| Lijiang | 0 | 0 | 0 | 0 | 0 | 0 | 0 | 0 | 0 | 0 | 0 | 31 |
| Pu'er | 0 | 0 | 0 | 2 | 0 | 1 | 0 | 159 | 0 | 0 | 0 | 1113 |
| Lincang | 0 | 0 | 0 | 90 | 0 | 244 | 4 | 228 | 0 | 0 | 12 | 1077 |
| Chuxiong | 0 | 0 | 0 | 0 | 0 | 0 | 0 | 0 | 0 | 0 | 0 | 29 |
| Honghe | 0 | 0 | 0 | 0 | 0 | 0 | 0 | 116 | 0 | 0 | 0 | 79 |
| Wenshan | 0 | 0 | 0 | 0 | 0 | 0 | 0 | 0 | 0 | 0 | 0 | 11 |
| Xishuangbanna | 3 | 1 | 1 | 1399 | 208 | 1659 | 675 | 3588 | 0 | 10 | 0 | 5385 |
| Dali | 0 | 0 | 0 | 0 | 0 | 0 | 0 | 8 | 0 | 0 | 0 | 64 |
| Dehong | 3 | 139 | 139 | 10 | 9 | 85 | 0 | 1149 | 237 | 0 | 505 | 3948 |
| Nujiang | 0 | 0 | 0 | 0 | 0 | 1 | 0 | 0 | 0 | 0 | 0 | 4 |
| Diqing | 0 | 0 | 0 | 0 | 0 | 0 | 0 | 0 | 0 | 0 | 0 | 6 |

Figure 2. Geographical distribution of dengue fever cases (B) Geographical distribution of imported dengue fever cases in Yunnan Province from 2012 to 2023.

| City/Prefecture | 2012 | 2013 | 2014 | 2015 | 2016 | 2017 | 2018 | 2019 | 2020 | 2021 | 2022 | 2023 |
| --- | --- | --- | --- | --- | --- | --- | --- | --- | --- | --- | --- | --- |
| Kunming | 4 | 27 | 18 | 29 | 27 | 39 | 71 | 298 | 8 | 3 | 2 | 34 |
| Qujing | 0 | 0 | 0 | 1 | 0 | 0 | 0 | 7 | 0 | 0 | 0 | 0 |
| Yuxi | 0 | 0 | 0 | 2 | 0 | 0 | 1 | 4 | 0 | 0 | 1 | 2 |
| Baoshan | 0 | 0 | 1 | 2 | 2 | 16 | 2 | 30 | 0 | 0 | 0 | 20 |
| Zhaotong | 0 | 0 | 0 | 0 | 0 | 0 | 0 | 3 | 0 | 0 | 0 | 1 |
| Lijiang | 0 | 1 | 0 | 0 | 0 | 0 | 0 | 0 | 0 | 0 | 0 | 0 |
| Pu'er | 1 | 0 | 0 | 1 | 0 | 2 | 2 | 33 | 0 | 0 | 0 | 47 |
| Lincang | 0 | 0 | 0 | 114 | 2 | 129 | 49 | 496 | 2 | 0 | 6 | 123 |
| Chuxiong | 0 | 0 | 0 | 0 | 0 | 0 | 0 | 2 | 0 | 0 | 0 | 0 |
| Honghe | 0 | 1 | 0 | 1 | 0 | 4 | 1 | 12 | 1 | 0 | 0 | 2 |
| Wenshan | 0 | 0 | 0 | 0 | 0 | 0 | 0 | 3 | 0 | 0 | 0 | 0 |
| Xishuangbanna | 29 | 34 | 0 | 85 | 30 | 203 | 63 | 291 | 2 | 0 | 0 | 920 |
| Dali | 0 | 0 | 0 | 0 | 0 | 0 | 0 | 6 | 0 | 0 | 0 | 0 |
| Dehong | 0 | 100 | 167 | 419 | 262 | 1158 | 75 | 346 | 15 | 1 | 41 | 267 |
| Nujiang | 0 | 0 | 0 | 0 | 1 | 0 | 0 | 0 | 0 | 0 | 0 | 0 |
| Diqing | 0 | 0 | 0 | 0 | 0 | 0 | 0 | 0 | 0 | 0 | 0 | 0 |

Figure 3. Countries of origin for imported dengue fever cases in Yunnan Province, China (A) Cases of countries of origin for imported dengue fever cases in Yunnan Province, China, from 2012 to 2023.

| Country | 2012 | 2013 | 2014 | 2015 | 2016 | 2017 | 2018 | 2019 | 2020 | 2021 | 2022 | 2023 |
| --- | --- | --- | --- | --- | --- | --- | --- | --- | --- | --- | --- | --- |
| Myanmar | 3 | 126 | 177 | 626 | 300 | 1497 | 186 | 1091 | 18 | 1 | 48 | 1298 |
| Laos | 27 | 24 | 0 | 6 | 7 | 37 | 3 | 146 | 3 | 1 | 0 | 107 |
| Cambodia | 0 | 0 | 0 | 0 | 1 | 0 | 52 | 246 | 3 | 0 | 0 | 2 |
| Thailand | 2 | 9 | 2 | 11 | 8 | 4 | 14 | 18 | 0 | 0 | 0 | 7 |
| Vietnam | 0 | 0 | 0 | 2 | 0 | 5 | 3 | 12 | 2 | 0 | 0 | 1 |
| Saudi Arabia | 0 | 0 | 0 | 0 | 0 | 0 | 0 | 0 | 0 | 0 | 0 | 1 |
| Papua New Guinea | 0 | 0 | 0 | 0 | 0 | 0 | 0 | 0 | 0 | 0 | 1 | 0 |
| Indonesia | 0 | 0 | 0 | 1 | 1 | 0 | 0 | 0 | 0 | 1 | 1 | 0 |
| Sri Lanka | 0 | 0 | 0 | 2 | 1 | 3 | 2 | 0 | 0 | 1 | 0 | 0 |
| Africa | 0 | 4 | 6 | 1 | 3 | 1 | 0 | 8 | 2 | 0 | 0 | 0 |
| Malaysia | 0 | 0 | 1 | 2 | 2 | 1 | 0 | 4 | 0 | 0 | 0 | 0 |
| Bangladesh | 0 | 0 | 0 | 3 | 0 | 1 | 1 | 2 | 0 | 0 | 0 | 0 |
| India | 2 | 0 | 0 | 0 | 1 | 1 | 0 | 1 | 0 | 0 | 0 | 0 |
| Maldives | 0 | 0 | 0 | 0 | 0 | 1 | 3 | 1 | 0 | 0 | 0 | 0 |
| Philippines | 0 | 0 | 0 | 0 | 0 | 0 | 0 | 2 | 0 | 0 | 0 | 0 |

Figure 3. Countries of origin for imported dengue fever cases in Yunnan Province, China (B) Source countries of imported dengue fever cases in Yunnan Province from 2012 to 2023

| Country | Province | Counts |
| --- | --- | --- |
| Myanmar | Yunnan Province | 5369 |
| Laos | Yunnan Province | 361 |
| Cambodia | Yunnan Province | 304 |
| Thailand | Yunnan Province | 180 |
| Vietnam | Yunnan Province | 25 |
| Saudi Arabia | Yunnan Province | 1 |
| Papua New Guinea | Yunnan Province | 1 |
| Indonesia | Yunnan Province | 4 |
| Sri Lanka | Yunnan Province | 8 |
| Africa | Yunnan Province | 25 |
| Malaysia | Yunnan Province | 10 |
| Bangladesh | Yunnan Province | 7 |
| India | Yunnan Province | 5 |
| Maldives | Yunnan Province | 4 |

Figure 3. Countries of origin for imported dengue fever cases in Yunnan Province, China (C) The top 5 countries with the most imported dengue fever cases to Yunnan Province, China, between 2012 and 2023

| Country | 2012 | 2013 | 2014 | 2015 | 2016 | 2017 | 2018 | 2019 | 2020 | 2021 | 2022 | 2023 |
| --- | --- | --- | --- | --- | --- | --- | --- | --- | --- | --- | --- | --- |
| Myanmar | 3 | 126 | 177 | 626 | 300 | 1497 | 186 | 1091 | 18 | 1 | 48 | 1298 |
| Laos | 27 | 24 | 0 | 6 | 7 | 37 | 3 | 146 | 3 | 1 | 0 | 107 |
| Cambodia | 0 | 0 | 0 | 0 | 1 | 0 | 52 | 246 | 3 | 0 | 0 | 2 |
| Thailand | 2 | 9 | 2 | 11 | 8 | 4 | 14 | 18 | 0 | 0 | 0 | 7 |
| Vietnam | 0 | 0 | 0 | 2 | 0 | 5 | 3 | 12 | 2 | 0 | 0 | 1 |

Figure 4. Comparison chart of predicted and actual imported dengue fever cases from 2020 to 2023

| Actual | | | | | | | | | | | | | Predicted | | | | |
| --- | --- | --- | --- | --- | --- | --- | --- | --- | --- | --- | --- | --- | --- | --- | --- | --- | --- |
|  | 2012 | 2013 | 2014 | 2015 | 2016 | 2017 | 2018 | 2019 | 2020 | 2021 | 2022 | 2023 |  | 2020 | 2021 | 2022 | 2023 |
| January | 1 | 5 | 0 | 2 | 4 | 3 | 6 | 15 | 7 | 0 | 0 | 0 |  | 5 | 30 | 26 | 42 |
| February | 1 | 0 | 2 | 2 | 4 | 1 | 2 | 6 | 2 | 0 | 0 | 0 |  | 7 | 25 | 27 | 39 |
| March | 2 | 2 | 0 | 0 | 1 | 0 | 1 | 7 | 1 | 0 | 2 | 1 |  | 11 | 26 | 30 | 39 |
| April | 0 | 0 | 1 | 1 | 0 | 7 | 1 | 10 | 1 | 0 | 0 | 0 |  | 14 | 28 | 32 | 41 |
| May | 0 | 0 | 3 | 1 | 2 | 2 | 3 | 49 | 1 | 0 | 0 | 0 |  | 21 | 54 | 39 | 58 |
| June | 1 | 4 | 2 | 9 | 4 | 15 | 8 | 77 | 0 | 1 | 0 | 6 |  | 29 | 72 | 45 | 71 |
| July | 4 | 6 | 3 | 57 | 10 | 81 | 16 | 109 | 0 | 0 | 0 | 100 |  | 38 | 93 | 53 | 85 |
| August | 4 | 22 | 22 | 68 | 8 | 199 | 36 | 163 | 2 | 1 | 0 | 455 |  | 56 | 130 | 68 | 109 |
| September | 9 | 43 | 44 | 145 | 47 | 313 | 46 | 419 | 1 | 1 | 5 | 566 |  | 86 | 296 | 102 | 219 |
| October | 7 | 59 | 64 | 236 | 110 | 551 | 58 | 396 | 9 | 1 | 24 | 214 |  | 92 | 282 | 105 | 210 |
| November | 2 | 21 | 38 | 112 | 114 | 296 | 60 | 233 | 4 | 0 | 17 | 62 |  | 79 | 176 | 87 | 141 |
| December | 3 | 1 | 7 | 21 | 20 | 83 | 30 | 47 | 0 | 0 | 2 | 13 |  | 43 | 54 | 53 | 60 |

Figure 5. Cumulative social network diagram and pathway diagram of imported dengue fever cases from 2012 to 2023

| Country | City/Prefecture | Counts |
| --- | --- | --- |
| Papua New Guinea | Kunming | 1 |
| Africa | Kunming | 25 |
| Cambodia | Baoshan | 1 |
| Cambodia | Chuxiong | 1 |
| Cambodia | Dali | 1 |
| Cambodia | Dehong | 19 |
| Cambodia | Honghe | 6 |
| Cambodia | Kunming | 229 |
| Cambodia | Lincang | 9 |
| Cambodia | Pu’er | 2 |
| Cambodia | Qujing | 6 |
| Cambodia | Wenshan | 3 |
| Cambodia | Xishuangbanna | 22 |
| Cambodia | Yuxi | 2 |
| Cambodia | Zhaotong | 3 |
| Laos | Baoshan | 1 |
| Laos | Dehong | 2 |
| Laos | Honghe | 2 |
| Laos | Kunming | 56 |
| Laos | Lincang | 2 |
| Laos | Pu’er | 6 |
| Laos | Xishuangbanna | 291 |
| Laos | Yuxi | 1 |
| Maldives | Kunming | 5 |
| Malaysia | Kunming | 8 |
| Malaysia | Pu’er | 1 |
| Malaysia | Yuxi | 1 |
| Bangladesh | Dehong | 1 |
| Bangladesh | Kunming | 5 |
| Bangladesh | Qujing | 1 |
| Myanmar | Baoshan | 71 |
| Myanmar | Chuxiong | 1 |
| Myanmar | Dali | 5 |
| Myanmar | Dehong | 2826 |
| Myanmar | Honghe | 5 |
| Myanmar | Kunming | 150 |
| Myanmar | Lijiang | 1 |
| Myanmar | Lincang | 910 |
| Myanmar | Nujiang | 1 |
| Myanmar | Pu’er | 74 |
| Myanmar | Qujing | 1 |
| Myanmar | Xishuangbanna | 1321 |
| Myanmar | Yuxi | 5 |
| Saudi Arabia | Kunming | 1 |
| Sri Lanka | Dehong | 1 |
| Sri Lanka | Honghe | 1 |
| Sri Lanka | Kunming | 7 |
| Thailand | Dehong | 2 |
| Thailand | Honghe | 1 |
| Thailand | Kunming | 47 |
| Thailand | Xishuangbanna | 23 |
| Thailand | Yuxi | 1 |
| Thailand | Zhaotong | 1 |
| India | Kunming | 5 |
| Indonesia | Kunming | 4 |
| Vietnam | Honghe | 7 |
| Vietnam | Kunming | 15 |
| Vietnam | Pu’er | 3 |
| Philippines | Kunming | 2 |
